# Patients’ willingness to pay for the treatment of tuberculosis in Nigeria: exploring own use and altruism

**DOI:** 10.1186/s12939-017-0574-2

**Published:** 2017-05-10

**Authors:** Ogbonnia G. Ochonma, Obinna E. Onwujekwe

**Affiliations:** 1 Department of Health Administration and Management, Faculty of Health Sciences and Technology, College of Medicine, University of Nigeria, Enugu Campus, Nsukka, Enugu State Nigeria; 2 Health Policy Research Group, Department of Pharmacology and Therapeutics, Faculty of Medical Sciences, College of Medicine, University of Nigeria, Enugu Campus, Nsukka, Enugu State Nigeria

**Keywords:** Tuberculosis, TB, Willingness to pay, Altruism, WTP, Nigeria

## Abstract

**Background:**

Although, current treatment services for Tuberculosis (TB) in Nigeria are provided free of charge in public facilities, the benefits (value) that patients attach to such service is not known. In addition, the prices that could be charged for treatment in case government and its partners withdraw from the provision of free services or inclusion of the services in health insurance plans are not known. Hence, there is a need to elicit the maximum amounts that patients are willing to pay for TB treatment services, both for themselves and for the very poor patients that may not be able to pay if some user fees are introduced (altruistic willingness to pay).

**Methods:**

A pretested interviewer-administered questionnaire was used to elicit the maximum willingness to pay (WTP) for TB treatment services from TB patients in a tertiary hospital in southeast Nigeria. WTP was elicited using the bidding game question format after a scenario was presented to the respondents. Data was analysed using tabulations. Tobit regression models were used to examine the validity of the elicited WTP for own use and altruistic WTP.

**Results:**

The results show that those aged 30 years and below constituted more than two-fifth (43.2%) of the respondents. More than half of the respondents (52.8%) were not employed. 100 (80.0%) of the respondents were willing to pay for their own use of TB treatment services while 78(62.4%) of the respondents were willing to make altruistic contributions so that the very poor could benefit from the TB services. A Tobit regression analysis of maximum WTP for TB for own use shows that respondents were willing to pay maximum amounts at different statistically significant levels. The results equally show that altruistic WTP was positively and statistically significantly related to the employment status, distance from UNTH and global seriousness of TB.

**Conclusions:**

Most patients positively valued the provision of free TB services and were willing to pay for TB treatment for own use. The better-off ones were also willing to make altruistic contributions. Free provision of TB treatment services is potentially worthwhile, but there is potential scope for continuation of universal provision of TB treatment services, even if the government and donors scale down their financing of the services.

**Electronic supplementary material:**

The online version of this article (doi:10.1186/s12939-017-0574-2) contains supplementary material, which is available to authorized users.

## Background

Tuberculosis (TB), a disease for which effective cure and preventive measures was discovered decades ago is still a major public health problem globally. It accounted for over a million deaths in 2010, with 95% of the deaths occurring in low and middle income countries [1]. An estimated US$ 8 billion per year is required to ensure a full response to the global tuberculosis (TB) epidemic: about two thirds for detection and treatment of drug susceptible TB. There is a funding gap of almost US$ 2 billion per year compared with the required total of US$ 8 billion [2].

In 2011, Nigeria with an estimated incidence of 133/100,000 population ranked ninth among the 22 countries that account for 80% of the TB burden in the world [3]. Also, 25% of TB patients in Nigeria are HIV co-infected [4].

The current level of government spending on healthcare in Nigeria is low, which limits the extent that it is able to fund priority public health services such as TB treatment services [5–7]. Presently, TB is being additionally funded through improved availability of Global Fund for AIDS, Tuberculosis and Malaria which increases the availability of health services for TB in Nigeria [5].

In Nigeria, treatment of TB is provided free of charge at public facilities and it is based on the directly observed treatment, short course (DOTS) strategy. The DOTS strategy requires that patients swallow their drugs under the direct observation of the health care workers at least in the first 2 to 3 months of treatment [8].

A major potential challenge for Nigeria is the sustainable financing of free TB services in view of current dwindling national income and international financial support, especially in the new quest to achieve universal health coverage (UHC) for TB treatment services. The problem of reduced funding and decrease of subsidies for TB is a possibility because of the contracting government revenues in Nigeria because of the dwindling price of crude oil. Hence, patients may have to bear some of the costs of treatment, if user fees are introduced for some or all of the TB treatment services. Therefore the aim of this study was to understand if patients would be willing to pay for TB services for own use and altruistically should the foreign donors reduce or cut off sponsorship in the face of limited and dwindling government total health expenditure.

Therefore, having an idea of the maximum amount of money that people are willing to pay for TB treatment is important for future cost-recovery of treatment from those that cannot afford and are willing to pay for such services. The payment may not be out-of-pocket but could be in form of insurance premiums in social health insurance plans. Also, it is important to know how patients value TB treatment services, which could provide an evidence-base for continuing government subsidies, especially taking into consideration the negative externalities of TB.

There is paucity of literature on information on willingness to pay (WTP) for tuberculosis services in sub-Saharan Africa, including Nigeria. However, in a study in New York City, the initial 24 subjects enrolled in a TB/HIV program, stated they would be willing to pay a median of $20 to get tested for HIV and $10 to get tested for TB [9]. Also a study on incremental cost effectiveness found that patients’ willingness to pay for a smear-positive TB case successfully treated increases with lowering the price of the testing for the patients. Patients were more willing to pay when the cost of the diagnosis and successful treatment were lowered [10].

However, the WTP technique elicited using the contingent valuation method (CVM) is increasingly been used to inform decision making in the health sector both globally and in Nigeria [11–15]. The CVM is a valid method for valuing benefits of goods and services where markets do not exist or the prices in markets do not reflect real prices [16]. WTP could be for own use, future use or for altruism [13]. The technique is frequently used in health, social and environmental programs for price setting and cost benefit analyses [17, 18]. It is relevant in determining WTP for TB treatment services because it incorporates the cost of pain and suffering, since people are expected to include them when evaluating how much they would pay to reduce their risk of illness or death [17, 19–23].

Hence, the study determined the level of WTP for TB treatment services by patients. It also explored the amounts of contributions that better-off patients are willing to pay so that very poor patients that are unable to pay will still receive treatment services (altruistic WTP). The information generated will be invaluable to decision makers as they plan for sustainability of TB treatment services in Nigeria.

This paper provides new policy relevant information on the value of benefits that TB patients attach to the free TB treatment services that they receive. The information is also relevant for pricing decisions to understand the scope for the continuation of provision of universal TB services if governments and donors scale down their funding of free TB treatment services.

## Methods

### Study area

The study was conducted in Enugu State, Southeast Nigeria. It is one of the 36 States that make up the Federal Republic of Nigeria. The State has a population of about 4.1 million people [23]. Over 90% of the inhabitants are *Igbos (a tribal group)* with about 90% of the population living in the rural areas [24]. Enugu State has 17 Local Government Areas with three Senatorial Zones – Enugu East, Enugu North and Enugu West Senatorial Zones [25, 26]. The State shares boundaries with 5 States of the Federation. It is bounded at the East and West by Ebonyi and Anambra States respectively. In the South, it has boundary with Abia State while in the north it has boundaries with Kogi and Benue State. Over 70% of the population that works is self-employed while rural dwellers are mainly peasant farmers or petty traders. Enugu lies within the tropical rain forest belt to the south. Most inhabitants (95%) are Christians while Muslims and traditionalists share the remaining 5% [27]. The native population is *Igbo* with few *Igala* near the borders with Kogi State (*Igbo* and *Igala* are two different tribal groups).

#### Study site

The University of Nigeria Teaching Hospital (UNTH) was purposively chosen as the site of the study because it is the major TB treatment centre not just for Enugu State but for all the adjoining States as described above. The hospital is well equipped and attended and has specialist nurses and physicians to attend to the public’s demand for TB services.

### Data collection

#### Sampling and sample size

The patients were all registered for TB treatment at the site and were at the time of the study undergoing TB treatment. All the patients (total sample frame) were eligible for inclusion in the study. The sampling method included all the patients registered at the TB treatment facility in the hospital. A total of 185 patients were registered with the TB treatment facility as at the time of this study. All the patients were given equal opportunity to be included in the study sample, however only 125 respondents representing 68% of the patient population at the facility responded by filling and returning the questionnaire. Data was collected in 2013.

### Sample size calculation/response rate

There was no sample size calculation in this study since all the patients (sample size) receiving treatment at the facility were all included in the study. There were 185 patients (sample size) receiving treatment at the facility as at the time of this study and were all included in the study. One hundred and twenty five (125) of the 185 patients were able to respond to our questionnaire. So we calculated the response rate by looking at the percentage of 125 of 185 which gave 67.56% and was rounded off to 68%.

### The data collection instrument--Questionnaire

A pre-tested interviewer-administered questionnaire was used to collect data on the following variables: patients’ socio-demographic data and their WTP for own use and for altruism. The questionnaire was divided into five sections. Section A: sought information on Health facility/pre-interview information while section B: concerned patient consent. Section C: was on patients’ general and demographic information and section D: assessed information on the objectives of the study and section E: concerned patients’ socio-economic factors. The questionnaire also explored the barriers to accessing TB services, the use of different health care providers/facilities before patients were diagnosed of TB at the treatment facility, the perception of seriousness of TB which examines patients’ understanding of how topical and damaging the TB disease is, the cost of accessing TB services which examined the monetary expenses in procuring TB services and the socio-economic status of the patients and their households.

### Ethical and distributional issues

The researchers were mindful of the ethical and distributional issues surrounding patients having to pay for the TB services since hitherto the services were being provided free of charge in Nigeria. They were made to understand that the programme was not for immediate implementation but to examine its possibility as this could always happen due to the dwindling economic fortune of Nigeria and shortage in global fund.

WTP was elicited using the bidding game question format. A scenario on paying for TB treatment services was presented to the respondents before their maximum levels of WTP was elicited. There were four Bidding Game (BG) iterations that examined whether the respondents will be willing to pay for the services they receive; how much they would be willing to pay for TB treatment per month; maximum amount they are willing to pay themselves; and maximum altruistic WTP:

### The iteration for eliciting WTP for own use of TB services (Additional file 1)

1. If you were to pay for the TB services you are now receiving, would you be willing to pay? [ ] 1 = yes 2 = No
2. What if the price you had to pay to avoid or prevent TB in the first place is higher than the amount you have said above, will you be willing to pay?1. [ ] 1 = yes 2 = no
3. What if the price you had to pay to avoid or prevent TB in the first place is lower than the amount you have said above, will you be willing to pay?[ ] 1. = Yes; 2 = no
4. Say, if due to inflation or an unforeseen economic situation, the price for TB services increased tremendously, what is the maximum amount you are very certain to pay bearing in mind your average monthly income. [ ] Allow respondent to say.
5. If you are not willing to pay any amount at all, what might be the possible reasons? [multiple answers are allowed]. 1 = yes 2 = no [ ] Do not believe my TB is curable; [ ] Do not have money; [ ] Do not want to continue on TB program; [ ] Do not like the TB services; [ ] My health is not getting any better

The iteration for eliciting Altruistic Willingness to Pay for TB Services

1. Are you willing to contribute extra N1, 000 per year so that those who could not afford TB services (due to say transportation, accommodation, extra medical purchases like vitamins costs etc.) could be provided with the services? [ ] 1 = yes 2 = no
2. What is the lowest amount you may be willing to pay so that TB services could be procured for the needy poor in your community? [ ] Naira.
3. What is the highest amount you may be willing to pay so that TB services could be procured for the needy poor in your community? [ ] Naira.
4. If you are not willing to pay the extra amount of money to help extend TB services to the poor, what could be your reasons? [multiple answers are allowed. []Do not believe in extending help to others; [ ] Do not have the money; [ ] Do not have a job;[ ] It is against my will and belief; [ ]It is against my culture; [ ]Others: (please explain)

### Pre-test and pilot test of the data collection instrument

The instrument was face validated by three researchers from the Faculty of Health Sciences and Technology, University of Nigeria, Enugu Campus. They were presented with the topic, purpose of the study, research questions and hypotheses of the study. They were requested to examine the entire items on the study instrument and determine their appropriateness, adequacy and clarity with reference to the purpose of the study, research questions and hypotheses. At the end of the validation, 63 variables were included in the instrument. The comments and observations of the validators formed the final draft of the instrument for this study.

In addition, the questionnaire was first pre-tested using a different set of patients from the study respondents (patients from the same clinic) 4 months earlier. This was done to measure patients’ understanding of the contents of the questions and to measure how the understanding of the questions were agreeable and same among the respondents and the researchers. Questions that were confusing and did not make any sense to the patients were either amended or discarded. Secondly, the questions were first translated into *igbo* (the *local language)* from English language *and* back to English language from *igbo* language to strengthen the content validity of the questions. Respondents who did not understand English language or the *igbo* language were interviewed using *pidgin English* language (local variance of English language). Finally, the reliability of the instrument was determined using the Conbach Alpha reliability estimate. The result of the reliability test showed a 0.85% for the pilot, indicating that the reliability of the instrument was high enough for the study.

### Data analysis (Additional file 2)

Tabulations of key variables were undertaken, with computation of means and standard deviations. Tobit regression was used to understand the factors that explained the maximum elicited WTP amounts. Tobit regression was chosen because the dependent variable had many zero responses and was hence a limited dependent variable. The independent variables in the regression analyses were factors that were a priori expected to explain WTP for TB services and included: respondents’ socio-demographic variables.

### Note: Exchange rate 1US$ = 178.57 Nigerian Naira

#### Ethics

Ethics approval for the study was obtained from the University of Nigeria Ethics Committee. In addition, informed consent was obtained from all the respondents. For example: “your consent is being sort before the commencement of this interview based on the Barriers to accessing and willingness to pay for tuberculosis (TB) diagnosis and treatment. Your anonymity will be protected as no particular mention of your name will be made in the process of the interview and in the analysis of this work thereafter”.

## Results

The result in Table 1 shows that those aged 30 years and below constituted more than two-fifth (43.2%) of the respondents. Sixty-three (50.4%) of the respondents were males. Only 4% of the respondents had no formal education, while 30.4%, 32.0% and 29% of respondents had primary, high school and college/university education respectively. More than half of the respondents (52.8%) were not employed. A little below fifty nine percent (58.4%) and 41.6% were married and single respectively.

**Table 1:** Socio-economic and demographic composition of the respondents

Socio demographic	Frequency N =125
	n (%)
Age group	
Under 30 years	54 (43.2)
31-40	25 (20)
41-50	29 (23.2)
Over 51	17 (13.6)
Gender	
Male	63 (50.4)
Female	62 (49.6)
Education level	
No school	5 (4.0)
Primary	38 (30.4)
High school	40 (32.0)
College/ University	37 (29.6)
Postgraduate	1 (0.8)
Literacy classes	4 (3.2)
Employment	
Employed	59(47.2)
Not employed	66 (52.8)
Marital Status	
Married	73 (58.4)
Single	52 (41.6)

Table 2 shows that 100 (80.0%) of the respondents were willing to pay for their own use of TB treatment services. The table also shows that 78(62.4%) of the respondents were willing to make altruistic contributions so that the very poor could benefit from the services. The average WTP by the respondents for their own use was US$178.48 whilst the average monthly altruistic WTP was US$14.06. The major reasons that people gave for being unwilling to pay were ‘do not have money’ 18 (90%) and ‘have no job’ 2 (10%).

**Table 2:** Number willing to pay and maximum WTP amounts for own use and for altruism

Variable	Measurement n (%)
Number willing to pay for own use:	100 (80%)
Number willing to pay N1000 altruism:	78 (62.4%)
WTP for own use	
Mean	$174.48
Standard Error	$25.80
Minimum - Maximum	$123.28–$225.68
WTP for altruism	
Mean	$14.06
Standard Error	$2.98
Minimum - Maximum	$8.15–$19.97
Reasons for not WTP	
Do not have money	18 (90)
Have no job	2 (10)

Table 3 is a Tobit regression analysis of maximum WTP for TB for own use. The result shows that respondents are willing to pay maximum amounts at different statistically significant levels. At 99% confidence interval, respondents were willing to contribute maximum amount for TB treatment for their own use. This was shown in respondents’ age, education, employment, marital status, global seriousness of TB, Seriousness of TB in Enugu and knowledge of patients about their TB status. When we measured the amount spent per month on TB, it was statistically significant at 95% CI. Other variables were not statistically significant at p.value less than 0.05. Coefficients of the regression were positively correlated at the measure of age, education, distance from UNTH, marital status and amount spent per month. Overall Tobit regression showed Chi2 value of 69.18 and p.value < 0.000.

**Table 3:** Tobit Regression Analysis of Maximum WTP for TB for own use

Maximum WTP	Coefficient	Standard Error	t-statistics	*p*-value
Age	16922.02	5855.03	2.89	0.005
Gender	-2898.624	9391.889	-0.31	0.758
Education	15929.44	4040.756	3.94	0
Employment	-32467.8	9853.953	-3.29	0.001
Distance from UNTH	3209.383	4302.664	0.458	0.458
Marital Status	8159.804	3245.418	0.014	0.014
Seriousness of TB (Global)	-33941.56	14070.76	-2.41	0.018
Seriousness of TB Enugu	-37850.43	11458.84	-3.3	0.001
Knowledge of patient’s TB Status	-40268.16	12384.72	3.25	0.002
Amount spend per month	19594.21	9450.879	2.07	0.041
Socioeconomic Status (SES)	-6423.262	4284.168	-1.5	0.137
Contant	67270.14	41782.99	1.61	0.111
Chi2 (10)				69.18
Prob.Chi2				0
Pseudo R2				0.031

The Tobit regression results on altruistic WTP is presented in Table 4. The results show that altruistic WTP was positively and statistically significantly related to the employment status, distance from UNTH and global seriousness of TB. However, the coefficients are negatively correlated with the age, distance from UNTH and perception of cost of treatment of TB. At 99% confidence interval, the analysis was only statistically significant with variable that represents distance from UNTH (0.003). The overall model was statistically significant with chi square value of 32.99, p. value <0.001 and Pseudo R2 of 0.015.

**Table 4:** Tobit regression on altruistic willingness to pay amounts

Maximum WTP	Coefficient	Standard Error	t-statistics	*p*-value
Age	-0.0103379	0.1634735	-0.06	0.95
Gender	0.1569221	0.2344318	0.67	0.505
Education	0.0255633	0.0977551	0.26	0.794
Employment	0.5420229	0.2624744	2.07	0.041
Distance from UNTH	-0.3476914	0.1125517	-3.09	0.003
Marital Status	0.0314625	0.0848673	0.37	0.712
Seriousness of TB (Global)	0.4847562	0.2270312	2.14	0.035
Seriousness of TB Enugu	0.2662743	0.2055335	1.3	0.198
Knowledge of patient’s TB Status	0.1713989	0.2837072	0.6	0.547
Perception of cost of treatment	-0.2740311	0.2326604	-1.18	0.242
Constant	0.0559046	0.1056758	0.53	0.598
Chi2 (10)				32.99
Prob.Chi2				0.001
Pseudo R2				0.0152

## Discussion

The findings show that majority of the respondents were willing to pay for TB services for own use. The relationships between many of the variables used in the willingness to pay and the Tobit regression models were consistent with demand theory and this suggests that findings were valid representation of peoples’ preferences. Such an association indicates that WTP is behaving as expected a priori, confirming the internal validity of the technique [18].

The finding that marital status was positively and statistically significantly related to WTP for owns use is consistent with anticipated demand theory. Those married are likely to be employed, have more disposable income and have greater valuable experience in economic decision making as they are likely to be older than the singles. Such an association means that WTP is behaving in a way expected of a priori [18, 20], thus affirming the construct validity of the findings. This result is consistent with [18, 21] where willingness to pay is associated with ability to pay.

The results also showed that some of the respondents were willing to pay for indigent community members to have access to TB treatment services. Hence, as reported by an earlier study in the study area [22], community solidarity through altruistic payments could be used to ensure that there is equity in access and use of TB treatment services by the poor, in the event that some user charges are introduced for such services.

The finding that a greater majority of the respondents were willing to contribute more towards own use of TB services compared to altruism also confirms economic expectation theory. Such an association indicates that WTP is also behaving as expected a priori [18, 20, 21]. It is theoretically expected that more respondents would be willing to pay for own use as against altruism. However, altruistic behaviour of patients should be encouraged through emphasis on the benefits derivable from TB control and the gains from less people being infected in the community which eventually may result into a free TB society.

Promoting patients altruistic behaviour could be brought to bear through various media, packaged to the understanding of the potential altruistic TB patients in consideration of their socio-demographic dispositions. The finding that altruism increased with higher attainment in educational level, distance from UNTH and global seriousness of TB probably shows that the more educated patients had more knowledge and appreciated the usefulness of treating TB amongst all respondents irrespective of socio-economic status. This is encouraging and consistent when considered in view of community-based health insurance (CBHI) schemes as in [23] where the better-off may actually contribute premiums for poor people with TB to benefit from treatment, if TB treatment services are included as part of the benefit package of CBHI schemes. Hence, the altruistic amounts elicited could be used to improve access to the most poor in a well managed TB programme by using the altruistic amounts of the rich in subsidizing the most poor.

Finally, the stated WTP amounts potentially mean that patients not only value the benefits of free treatment, but were willing to pay for TB services for own use. This will improve the chances of the TB programme to be sustained in the event the Nigerian government and international community reduced or stopped funding for TB treatment. Also the fact that altruism was equally high is encouraging as the amounts elicited could be used to improve access to the most poor in a well managed TB programme in the event patients may have to pay whole or part of the TB services in the event international donors and the government would withdraw TB sponsorship.

Equally encouraging for the sustainability of the TB programme, is the fact that the employed were willing to make more altruistic contribution to benefit the poor indicating that an improved Nigerian economy in the future may actually benefit the sustainability of the TB programme in the event of government and international donor reduced or stoppage of TB funding.

### Implications of the study's results for public health policies

Based on analysis of current situation of TB in Nigeria and in line with internationally-recommended standards, six strategic approaches have been adopted by Nigeria Stop TB Partnership aimed at reducing the burden of TB [28]. Of particular importance and relevance to our research findings is strategy two: addressing the needs of the poor and vulnerable population and empowering household and communities especially the poor and vulnerable groups to provide TB care and control services. This strategy could be boosted through encouraging altruistic payment of TB services by the financially better off among TB patients to benefit the very poor. A wider research investigating altruism in TB services payment is encouraged to determine the extent of the altruistic behaviour so as to be included in the TB payment arrangements to ensure equity in the event individuals would have to pay for TB services in the future.

## Conclusions

Our study has shown that most patients are willing to pay for TB services both for own use and for altruism. It shows that investing both government and donor funds in providing free TB treatment services is worthwhile and provides value for money. However, if such funding is constrained, it will be possible to introduce a form of co-financing so that TB treatment services are not disrupted. Also, decision makers and actors should explore the possibility of using altruistic WTP in filling the funding gaps expected in the future financing of TB control and to bring equity in service utilization among the different SES respondents.

### Study limitation

We do caution that our study has some limitations amongst which is the fact that it may not be generalizable to the general population in Nigeria in that it was limited in scope as only one DOTS clinic was involved. Other than that, its finding could be approximated to similar population settings in Nigeria and it is the first of its kind in country. Equally, the questionnaire used in this study has high validity and reliability as it was developed and used severally by the World health organization in TB studies.

## Additional files

Additional file 1: The Questionnaire. (DOC 91 kb)

Additional file 2: The SPSS data entry. (SAV 203 kb)

## Abbreviations

AWTP: Altruistic willingness to pay
BG: Bidding Game
CBHI: Community-based health insurance
CVM: Contingent valuation method
HIV: Human immune virus
MTL: Malaria, Tuberculosis and Leprosy
SES: Socio Economic Status
TB: Tuberculosis
UHC: Universal health coverage
UNTH: University of Nigeria Teaching Hospital
WTP: Willingness to pay
