# Supplementary material for: Patients’ willingness to pay for the treatment of tuberculosis in Nigeria: exploring own use and altruism
Source: Int J Equity Health. 2017 May 10;16:74. doi: 10.1186/s12939-017-0574-2 (PMC5424411; doi:10.1186/s12939-017-0574-2)
Supplement: Supplementary file 1 — The Questionnaire. (DOC 91 kb) [file 12939_2017_574_MOESM1_ESM.doc]

**Directions:** Please, I am a student and a faculty member in the Department of Health Administration and Management, studying in the MSc Program--Health Economics, Management and Policy. I am about completing my program with a research on Patients’ Willingness to pay for Tuberculosis Diagnosis and Treatment. Please, I do solicit your cooperation in answering to the best of your knowledge the questions below bordering on the possible barriers you may have encountered in the process of obtaining TB Services. The questions will also solicit your answers relating to your willingness to paying for the TB Services. In this work, Your Willingness to Pay for TB Services will be determined by the monetary values you may place on the Services if you were to pay for them. The questions will require fifteen (15) minutes of your time and your anonymity is protected.

**INSTRUCTION:** Enumerator, please fill sections **A and B** at the beginning of the interview on the health facility/Pre-interview information and consent respectively.

**SECTION A: Health facility/pre-interview information**

**Name of the facility/clinic interview was held: ------------------------------**

**What is the date of the interview: --------------------------------------------------**

**What time did the interview start: -------------------------------------------------**

**What time did the interview end: --------------------------------------------------**

**Respondent Code: ----------------------------------------------------------------**

**Name of the interviewer: ------------------------------------------------------**

**Signature of the interviewer: -------------------------------------------------**

**SECTION B: Consent**

**ENUMERATOR:** Please read out the consent information to the patient and allow for his/her signature before proceeding.

**YOUR CONSENT** is being sort before the commencement of this interview based on the Barriers to accessing and willingness to pay for tuberculosis (TB) diagnosis and treatment among different population groups in Enugu State, Nigeria.

A questionnaire has been structured to solicit the information pertaining to the above research topic and you will be guided as the interview continues.

Your anonymity will be protected as no particular mention of your name will be made in the process of the interview and in the analysis of this work thereafter.

**PATIENT SIGNATURE------------------------------------------------------------**

**INTERVIEWER SIGNATURE----------------------------------------------------**

**RESEARCH PROPOSAL TITLE:**

**Barriers to accessing and willingness to pay for tuberculosis (TB) diagnosis and treatment among different population groups in Enugu State, Nigeria.**

**SECTION C: General information**

- **Please as appropriate. General and Demographic Questions:**

1. How old are you? [2]

1. [ ] under 30

2. [ ] 31 – 40

3. [ ] 41 – 50

4. [ ] over 50

2. What is your gender? [3]

1. [ ] Male

2. [ ] Female

1. What is the highest level of education you have completed? [4]
2. [ ] No school
3. [ ] Elementary
4. [ ] High school
5. [ ] College/University
6. [ ] Higher education (professional or post-graduate)
7. [ ] Religious schooling only
8. [ ] Literacy classes only.
9. Do you currently have paid employment? [5]
10. [ ] Yes
11. [ ] No
12. How far do you live from the UNTH (TB) health clinic? [6]

1. [ ] 0 - 10 kilometres

2. [ ] 11 – 20 kilometres

3. [ ] 21 – 30 kilometres

4. [ ] More than 30 kilometres.

1. Marital Status [7]
2. [ ] Married
3. [ ] Separated
4. [ ] Divorced
5. [ ] Married with Children
6. [ ] Married without Children
7. [ ] Single

**SECTION D: Assessing the objectives**

**Objective 1: To determine the different barriers to accessing TB diagnosis and treatment in Enugu State, Nigeria.**

**Health-Seeking behaviour:**

1. Where do you usually go if you are sick, or to treat a general problem?

(Check all that are mentioned.)

1. [ ] Private clinic [7a] [8]

2. [ ] Government clinic or hospital [7b] [9]

3. [ ] Traditional or homeopathetic healer [7c] [10]

4. [ ] Clinic run by a non-governmental organization [7d] [11]

5. [ ] Faith houses and Church [7e] [12]

6. [ ] Other: [7f] [13]

8. How often do you generally seek health care at a clinic or hospital? (Check one)[14]

1. [ ] Twice a year or more

2. [ ] Once per year

3. [ ] Less than once a year but at least twice in past 5 years

4. [ ] Once in past 5 years.

5. [ ] Never in past 5 years.

6. [ ] Other: [15]

**TB Knowledge and Awareness**

9.Where did you first learn about tuberculosis (TB)?

(Check all that are mentioned.)

1. [ ] Newspapers and magazines [9a] [16]
2. [ ] Radio [9b] [17]
3. [ ] TV [9c] [18]
4. [ ] Billboards [9d] [19]
5. [ ] Brochures, posters and other printed materials[9e] [20]
6. [ ] Health workers [9f] [21]
7. [ ] Family, friends, neighbours and colleagues.[9g] [22]

10. In your opinion, how serious a disease is TB? [23]

(Check one)

1. [ ] Very serious
2. [ ] Somewhat serious
3. [ ] Not very serious

11. How serious a problem do you think TB is in the country or Enugu State? (Check one) [24]

1. [ ] Very serious

2. [ ] Somewhat serious

3. [ ] Not very serious

12. What are the signs and symptoms of TB?

(Please check all that are mentioned.)

1. [ ] Rash [12a] [25]

2. [ ] Cough [12b] [26]

3. [ ] Cough that last longer than 3 weeks [12c] [27]

4. [ ] coughing up blood [12d] [28]

5. [ ] severe headache [12e] [29]

6. [ ] Nausea [12f] [30]

7. [ ] Weight loss [12g] [31]

8. [ ] Fever [12h] [32]

9. [ ] Fever without clear cause that lasts more than 7 days. [12i] [33]

10. [ ] Chest pain [12J] [34]

11. [ ] Shortness of breath [12k] [35]

12. [ ] Ongoing fatigue [12L] [36]

13. [ ] Do not know [12M] [37]

14. [ ] Other [12N] [38]

13. How can a person get TB? (Please check all that are mentioned.)

1. [ ] Through handshakes [13a] [39]

2. [ ] Through the air when a person with TB coughs or sneezes [13b] [40]

3. [ ] Through sharing dishes [[13c] [41]

4. [ ] Through eating from same plate [13d] [42]

5. [ ] Through touching items in public places (doorknobs, handles in transportation, etc) [13e] [43]

6. [ ] do not know [13f] [44]

7. [ ] Other (please explain): [45] [specify] [46]

14. How can a person prevent getting TB? (Please check cell that are mentioned.)

1. [ ] Avoid shaking hands [14a] [47]

2. [ ] Covering mouth and nose when coughing or sneezing. [14b] [48]

3. [ ] Avoid sharing dishes [14c] [49]

4. [ ] washing hands after touching items in public places [14d] [50]

5. [ ] Closing windows at home [14e] [51]

6. [ ] Through good nutrition [14f] [52]

7. [ ] By praying [14g] [53]

8. [ ] Do not know [14h] [54]

9. [ ] Other (please explain): [55]

15. In your opinion, who can be infected with TB? (Please check all that are mentioned.

1. [ ] Anybody [15a] [56]

2. [ ] Only poor people [15b] [57]

3. [ ] Only homeless people [15c] [58]

4. [ ] Only alcoholics [15d] [59]

5. [ ] Only drug users [15e] [60]

6. [ ] Only people living with HIV/AIDS [15f] [61]

7. [ ] Only people who have been in prison [15g] [62]

8. [ ] Other (please explain): [63]

16. Can TB be cured? [64]

1. [ ] Yes

2. [ ] No

17. How can someone with TB be cured? (Check all that are mentioned)

1. [ ] Herbal remedies [17a] [65]

2. [ ] Home rest without medicine [17b] [66]

3. [ ] Praying [17c] [67]

4. [ ] Specific drugs given by health centre [17d] [68]

5. [ ] DOTS [17e] [69]

6. [ ] Do not know [17f] [70]

7. [ ] Other: [71]

**TB Attitudes and Care-Seeking Behaviour**

18. Did you ever think you could get TB? (Ask respondents to please explain his/her answer) [72]

1. [ ] Yes (because) [73]

2. [ ] No (because) [74]

19. What was your reaction when you found out that you had TB? (Check all that are mentioned.)

1. [ ] Fear [19a] [75]

2. [ ] Surprise [19b] [76]

3. [ ] Share [19c] [77]

4. [ ] Embarrassment [19d] [78]

5. [ ] Sadness or hopelessness [19e] [79]

6. [ ] Other: [80]

20. Who were you able to talk to you, when you found out you had TB? (Check all that are mentioned.)

1. [ ] Doctor or other medical worker [20a] [81]

2. [ ] Spouse [20b] [82]

3. [ ] Parent [20c] [83]

4. [ ] children [20d] [84]

5. [ ] Other family member [20e] [85]

6. [ ] Close friend [20f] [86]

7. [ ] No one [20e] [87]

8. [ ] other: [88]

21. What did you do when you thought you had symptoms of TB? (Check all that apply.)

1. [ ] Went to health facility [21a] [89]

2. [ ] Went to pharmacy [21b] [90]

3. [ ] Went to traditional healer [21c] [91]

4. [ ] pursued other self-treatment options (herbs, etc.) [21d] [92]

5. [ ] Other: [93]

22. When you found out that you had symptoms of TB, at what point did go to the health facility? [94]

1. [ ] when treatment on my own did not work > go to QH23

2. [ ] When symptoms that looked like TB lasted for 3 – 4 weeks > go to QH23

3. [ ] went as soon as I realized that my symptoms might be related to TB > go to QH23

4. [ ] I never went to the related doctor > go to QH23

23. If you never went to the doctor/health facility, what was the reason?

1. [ ] Not sure where to go [23a] [95]

2. [ ] Cost [23b] [96]

3. [ ] Difficulties with transportation/distance to clinic

[23c] [97]

4. [ ] Did not trust medical workers [23d] [98]

5. [ ] Did not like the attitude of medical workers [23e] [99]

6. [ ] Could not leave work (overlapping work hours with medical facility working hours) [23f] [100]

7. [ ] Did not want to find out that something was really wrong [23g] [101]

8. [ ] Other (please explain): [23h] [102]

24. How expensive do you think the TB diagnosis and treatment is in this country/state—consider all other costs including transportation, lodging, purchase of non-provided vitamins and medications? [103]

1. [ ] It is free of charge

2. [ ] It is reasonably priced

3. [ ] It is somewhat/moderately expensive

4. [ ] It is very expressive.

Interviewer: If respondent gives monetary amount, note the amount here: [104]

**TB Attitudes and Stigma**

25. Do people now know you have TB? [105]

1. [ ] Yes

2. [ ] No

26. Which statement is closest to your feeling now that people know about your TB status?

1. [ ] “They feel compassion and desire to help [26a] [106]

2. [ ] “They feel compassion but they tend to stay away from me” [26b] [107]

3. [ ] “They feel it is your problem and they cannot get

TB” [26c] [108]

4. [ ] “They fear me, because I may infect them” [26d] [109]

5. [ ] “They have no particular feeling.” [26e] [110]

6. [ ] Other (please explain): [26f] [111]

27. In your community, how is a person who has TB usually regarded/treated?

1. [ ] Most people reject him or her [27a] [112]

2. [ ] Most people are friendly, but they generally try to avoid him or her [27b] [113]

3. [ ] The community mostly supports and helps him or her [27c] [114

4. [ ] Other (please explain): [27d] [115]

28. Do you think that HIV positive people should be concerned about TB? [116]

28a) Why [117]

1. [ ] Person with HIV is more likely to develop TB

2. [ ] Do not know

3. [ ] Other (please explain): [118]

28b) Why not [119]

1. [ ] Person with HIV is not more likely than person without HIV to develop TB

2. [ ] do not know

3. [ ] Other (please explain): [120]

**TB Awareness and Source of Information**

29. Do you feel well informed about TB? [121]

1. [ ] Yes

2. [ ] No

30. Do you wish you could get more information about TB? [122]

1. [ ] Yes

2. [ ] No

31. What are the sources of information that you think can most effectively reach people like you with information on TB? (Please choose the three most effective sources.)

1. [ ] Newspapers and magazines [31a] [123]

2. [ ] Radio [31b] [124]

3. [ ] Billboards [31c] [125]

4. [ ] TV [31d] [126]

5. [ ] Brochures, posters and other printed materials [31e] [127]

6. [ ] health workers [31f] [128]

7. [ ] Family, friends, neighbours and colleagues [31g] [129]

8. [ ] Religious leaders [31h] [130]

9. [ ] Teachers [31i] [131]

10. [ ] Other (please explain): [31j] [132]

32. What worries you the most now that you know you have TB? [133]

1. [ ] If I will ever get cured

2. [ ] The rejection of my family by neighbours and relatives

3. [ ] My rejection by friends, colleagues and relatives

4. [ ] The rejection imposed by my culture

5. [ ] The associated costs in procuring TB services

6. [ ] What happens to my family in the event of my death

7. [ ] Other (please explain):- [32b] [134]------------------------------------

**Objective 2: To access the Use of Different Healthcare Providers for the Diagnosis and Treatment of Tuberculosis in Enugu State, Nigeria.**

**Where did Patients First Go for Diagnosis**

33. When the symptoms or signs of TB presented themselves, who did you confide in? [135]

1. [ ] Spouse

2. [ ] A family member

3. [ ] A friend

4. [ ] A distant relative

5. [ ] Other (please explain): [33b] [136]

34. What was the first place you went to seek for help? [137]

1. [ ] Herbal remedies

2. [ ] DOTS/Health centres

3. [ ] Did not have an idea on what to do

4. [ ] Spiritual houses/healers

5. [ ] Other (please explain): [34b] [138]

35. Where were you actually diagnosed with TB? [139]

1. [ ] Herbal house

2. [ ] Native doctor

3. [ ] DOTS/Health centre

4. [ ] Spiritual house/church

5. [ ] Other (please explain): [35b] [140]

36. Who directed you to the place where you were actually diagnosed with TB? [141]

1. [ ] Spouse

2. [ ] Friend

3. [ ] Family members

4. [ ] Went on my own accord

5. [ ] Other (please explain): [36b] [142]

**Patient TB Treatment**

37. Now that you have been diagnosed with TB, how are you finding your treatment? [143]

1. [ ] Have no difficulty with treatment

2. [ ] Have little difficulty with treatment

3. [ ] Have moderate difficulty with treatment

4. [ ] Have most difficulty with treatment

5. [ ] May stop treatment in due course due to treatment problems.

38. What is the difficulty associated with your TB treatment.

1. [ ] Personal problem - due to either distance or financial problem [38a] [144]

2. [ ] Institutional/family problem [38b] [145]

3. [ ] Stigma – peoples’ attitude towards you [38c] [146]

4. [ ] Do not believe my TB problem has a cure [38d] [147]

5. [ ] My condition is not getting any better. [38e] [148]

39. How often do you travel to the health facility/hospital for taking your TB drugs? [149]

1. [ ] Every two weeks

2. [ ] Every month

3. [ ] Every two months

4. [ ] Every three months

5. [ ] Every four months or more.

**Patient Indirect Costs before and During Diagnosis**

40. Have you ever stopped working/doing house work due to TB? [150]

1. [ ] Yes

2. [ ] No

41. If the answer to the above is yes, what was your estimated personal take home earnings per month BEFORE the TB illness?

Allow respondent to state the amount [151]

42. If you employed someone to do housework for your household, how much would you have to pay him/her per day?

1. While you are sick [42a] [152]

2. While you are healthy 42b] [153

Allow respondent to state the amount.

**Direct Costs of Patients During Treatment**

43. How often do you travel to the health facility/hospital for taking your TB drugs/check ups? [154]

1. [ ] Every two weeks or less

2. [ ] Every month

3. [ ] Every two months

4. [ ] Every three months

5. [ ] every four months or more.

44. From your home to the facility, how much does it cost if you take a transport to and fro? [155]

1. [ ] One thousand Naira (N1, 000) or less

2. [ ] Between two thousand Naira and three thousand Naira (N2, 000 - N3, 000)

3. [ ] Between four and five thousand Naira (N4, 000 –

N5, 000)

4. [ ] Six thousand Naira (N6, 000)

5. [ ] More than six thousand Naira (N6, 000^+^)

45. If you go to a facility, how much do you spend on food on that day? [156]

1. [ ] Five hundred Naira or less (N500^-^)

2. [ ] Five hundred Naira or more (N500^+^)

3. [ ] One thousand Naira (N1, 000)

4. [ ] One thousand five hundred Naira or more (N1, 500^+^)

5. [ ] Two thousand Naira or more (N2, 000^+^)

46. Do you have to pay administration fees when picking up your TB drugs /check ups? [46a] [157]

1. [ ] Five hundred Naira or less (N500^-^)

2. [ ] Five hundred Naira or more (N500^+^)

3. [ ] One thousand Naira (N1, 000)

4. [ ] One thousand five hundred Naira or more (N1, 500^+^)

5. [ ] Two thousand Naira or more (N2, 000+) [158]

47. Do you have any accommodation costs when picking up your TB drug/check ups? [159]

1. [ ] One thousand Naira or more (N1, 000^+^)

2. [ ] Between one thousand five and two thousand Naira (N1, 500 – N2, 000)

3. [ ] Two thousand Naira or more (N2, 000^+^)

4. [ ] Between two thousand five hundred Naira and three thousand Naira (N2, 500 – N3, 000)

5. [ ] Three thousand Naira or more (N3, 000^+^)

48. Do you buy any supplements for your diet because of the TB illness, for example vitamins, energy drinks, soft drinks, fruits or medicine? [160]

1. [ ] Yes

2. [ ] No

49. How much did you spend on these items last month approximately? [161]

1. [ ] five hundred Naira or less (N500^-^)

2. [ ] Between five hundred Naira and one thousand Naira (N500 – N1, 000)

**Objective 3: To quantify the willingness to pay (WTP) and the maximum amounts tuberculosis positive individuals are prepared to pay for treatment.**

**Willingness and Ability to Pay**

50. If you were to pay for the TB services you are now receiving, would you be willing to pay? [162]

1. [ ] Yes

2. [ ] No

51. What is your average mostly income?

Allow respondent to say. [163]

1. [ ] Have no income

2. [ ] N10, 000 – N18, 000

3. [ ] N18, 100 – N30, 000

4. [ ] N30, 100 – N50, 000

5. [ ] N50, 100 – N100, 000

6. [ ] N100, 100 – N200, 000

7. [ ] N200, 100 and above

52. How much would you be willing to pay for not becoming ill with TB? [164]

Allow respondent to say.

53. What if the price you had to pay to avoid or prevent TB in the first place is higher than the amount you have said above, will you be willing to pay? [165]

1. [ ] Yes

2. [ ] No

54. What if the price you had to pay to avoid or prevent TB in the first place is lower than the amount you have said above, will you be willing to pay? [166]

1. [ ] Yes

2. [ ] No

55. Say, if due to inflation or an unforeseen economic situation, the price for TB services increased tremendously, what is the maximum amount you are very certain to pay bearing in mind your average monthly income. [167]

Allow respondent to say.

56. If you are not willing to pay any amount at all, what might be the possible reasons? [168]

1. [ ] Do not believe my TB is curable.

2. [ ] Do not have money

3. [ ] Do not want to continue on TB program

4. [ ] Do not like the TB services

5. [ ] My health is not getting any better

**Objective 4: To verify whether these patients are also willing to pay for indigent community members who may be afflicted with TB but do not have the ability to afford its services (altruism).**

**Altruistic WTP for TB Services**

It has been a known fact that some people who are too poor may not have the money to pay for their TB services, but really do need the services to improve on their health status and productivity. An organization is to be set up to collect and handle extra cash and to apply it to judicious use and see how the extremely poor could benefit from it.

57. Are you willing to contribute extra N1, 000 per year so that those who could not afford TB services (due to say transportation, accommodation, extra medical purchases like vitamins costs etc.) could be provided with the services? [169]

1. [ ] Yes

2. [ ] No

58. What is the lowest amount you may be willing to pay so that TB services could be procured for the needy poor in your community? [170]

[ ] Naira.

59. What is the highest amount you may be willing to pay so that TB services could be procured for the needy poor in your community? [171]

[ ] Naira.

60. If you are not willing to pay the extra amount of money to help extend TB services to the poor, what could be your reasons? [60a] [172]

1. [ ] Do not believe in extending help to others

2. [ ] Do not have the money

3. [ ] Do not have a job

4. [ ] It is against my will and belief

5. [ ] It is against my culture

6. [ ] Others: (please explain) [173]

**SECTION F: Patient Socio-Economic Factors.**

61**. FOOD/OTHER BILLS:** How much money did you and your household spend purchasing **food and the following** items in the past month.

1. Food/Feeding-------------------N [ ] [61a] [174]
2. Monthly Electricity bill--------N [ ] [61b] [175]
3. Laundry-------------------------N [ ] [61c] [176]
4. Transportation and Vehicular Maintenance N[ ][61d] [177]
5. Water bill-----------------------N [ ] [61e] [178]
6. News Paper, Cable TV bill----N [ ] [61f] [179]
7. Cooking Gas/firewood/kerosene-N[ ] [61g][180]
8. Other (specify)-----------------N [ ] [61h] [181]
9. **Total-------------------------------N** [ ] [61i] [182]

62**. ASSETS:** From the **assets** that I am about reading out; please indicate which of them that is functional that you and your household have. Yes=1, No=0

1. Radio [ ] [62a] [183]

2. Bicycle [ ] [62b] [184]

3. Motor car [ ] [62c] [185]

4. Television set [ ] [62d] [186]

5. Gen. Set [ ] [62e] [187]

6. Air conditioner [ ] [62f] [188]

63. **HOUSE/RENT:** Do you rent where you are presently living or do you own the house you are presently living in. Yes=1, No=0 [189]

THANK YOU.
